# Normalized difference vegetation index sensor-based nitrogen management in bread wheat (*Triticum aestivum* L.): Nutrient uptake, use efficiency, and partial nutrient balance

**DOI:** 10.3389/fpls.2023.1153500

**Published:** 2023-04-04

**Authors:** Biplab Mitra, Prantick Singha, Arnab Roy Chowdhury, Abhas Kumar Sinha, Milan Skalicky, Marian Brestic, Saud Alamri, Akbar Hossain

**Affiliations:** ^1^ Department of Agronomy, Uttar Banga Krishi Viswavidyalaya, Coochbehar, West Bengal, India; ^2^ Department of Soil Science and Agricultural Chemistry, Uttar Banga Krishi Viswavidyalaya, Coochbehar, West Bengal, India; ^3^ Department of Botany and Plant Physiology, Faculty of Agrobiology, Food, and Natural Resources, Czech University of Life Sciences Prague, Prague, Czechia; ^4^ Institute of Plant and Environmental Sciences, Slovak University of Agriculture, Nitra, Slovak; ^5^ Department of Botany and Microbiology, College of Science, King Saud University, Riyadh, Saudi Arabia; ^6^ Division of Soil Science, Bangladesh Wheat and Maize Research Institute, Dinajpur, Bangladesh

**Keywords:** wheat, NDVI sensor, variable nitrogen doses, partial nutrient balance, nitrogen uptake, nitrogen use efficiency

## Abstract

The present experiment was conducted to assess the impact of fixed and variable doses (using a normalized difference vegetation index-sensor) of nitrogen (N) on wheat yields, nutrient uptake, nitrogen use efficiency, and soil nitrogen balance through the optimization of nitrogen dose. There were 10 treatments based on fixed and variable doses with different splits, and each treatment was replicated three times under a randomized complete block design. The treatments comprised fixed doses of 120 and 150 kg N ha^–1^ with different splits; variable doses based on sensor readings after application of 60, 90, and 120 kg N ha^–1^; 225 kg N ha^–1^ as a nitrogen-rich control; and no application of nitrogen as the absolute control. It was revealed that the application of a basal dose of 60 kg N ha^–1^ and another 60 kg N ha^–1^ at the crown root initiation stage followed by a sensor-guided N application significantly improved wheat grain yields and grain nitrogen uptake. However, straw nitrogen uptake was highest in N-rich plots where 225 kg N ha^–1^was applied. It was found that any curtailment in these doses at basal and crown root initiation stages followed by nitrogen application using a normalized difference vegetation index sensor later could not bring about higher crop yields. On average, wheat crops responded to 152–155 kg N ha^–1^ in both years of the study. Partial factor productivity along with agronomic and economic nitrogen use efficiency showed a declining trend with an increased rate of N application. Apparent N recovery values were comparable between normalized difference vegetation index sensor-based N application treatments and treatments receiving lesser N doses. Soil N status decreased in all the treatments except the nitrogen-rich strip, where there was a marginal increase in soil N status after the wheat crop harvest in the rotation. Partial nitrogen balance was negative for all the treatments except the control. From these 2-year field trials, it can be concluded that applying a normalized difference vegetation index sensor could be an essential tool for the rational management of fertilizer nitrogen in wheat grown in eastern sub-Himalayan plains.

## 1 Introduction

Sustainable crop production remains a significant challenge, and it has drawn increasing attention from scientific communities. The alarming rate of natural resource degradation is a substantial concern for moving forward in agriculture, specifically in developing countries (Clair and Lynch, 2010). The present cultivation practices in the rice–wheat system are degrading the soil and water resources, and thus threatening the sustainability of the system (Chauhan et al., 2012; Kumar et al., 2018). The crop production system is nutrient dependent; in the last five decades, nutrient applications have significantly improved crop yields. However, owing to input mismanagement, biodiversity, soil quality, and air quality have been badly damaged, resulting in a negative impact on the environment (Godfray and Garnett, 2014). However, ideal crop management should always aim to enhance nutrient use efficiency, particularly that of nitrogen (N) as higher nitrogen use efficiency (NUE) is observed chiefly with lower rates (Majumdar et al., 2013; Mondal et al., 2018). Without compromising productivity and profitability, using the optimum quantity of nutrients is vital for agricultural sustainability (Salim and Raza, 2020). That is why to achieve sustainability, yields, economics, and nutrient use efficiency need to be considered with equal weighting (Hulmani et al., 2022).

The rice–wheat (R–W) cropping system occupies around 24 million hectares in Asian sub-tropical countries, including India, China, Bangladesh, China, and Nepal (Singh et al., 2013). The system occupies over 6.22 million hectares in the eastern Gangetic plains alone (Timsina et al., 2018). Most of the farmers in this region are continuing to intensify the cropping system by growing wheat during winter months, owing to high subsidies for agricultural inputs such as power, irrigation water, fertilizer, etc. (Saharawat et al., 2009). However, the rice–wheat system across southern Asia is water-, energy-, capital-, and labor-intensive and becomes less profitable as the availability of resources diminishes (Bhatt et al., 2016). Owing to poor soil fertility status, water and temperature stress, significant pest and disease infestation, groundwater depletion, escalating production costs, labor scarcity, climatic vulnerabilities, and, more importantly, deteriorating soil health caused by imbalanced fertilization, crop yields are suffering and sustainability issues are worsening (Islam et al., 2019; Dhanda et al., 2022). Sub-optimal nutrient management vis-à-vis nutrient mining in these tracts has resulted in production fatigue with poor nutrient use efficiency (Dhawan et al., 2021).

In the entire Indo-Gangetic Plain, wheat is mostly grown after monsoon rice during the dry winter season, with 100% of phosphorous (P) and potassium (K) doses and 50% of N doses being applied before final land preparation. Depending on irrigation events, the remaining N is applied in one or two splits before the maximum tillering stage (Pathak et al., 2006). N fertilizer in wheat is mostly applied in blanket doses without any consideration of existing soil fertility status. The inefficient use of N fertilizers results in poor NUE in wheat grown in the region (Adhikari et al., 1999; Dobermann et al., 2003). To a great extent, NUE suffers because of the large-scale blanket application of fertilizers, particularly nitrogen fertilizers (Majumdar et al., 2013; Mitra et al., 2019). Splitting the N doses might be considered an efficient tool through which NUEs could be significantly increased compared with blanket fertilizer application (Khan and Akma, 2021; Derebe et al., 2022). Inefficient splitting of N doses along with excess N applications may be the reason for lower NUEs; however, controversies still exist regarding N fertilization and maximizing yield with improved NUE in wheat production.

Real-time nitrogen management tools, such as a leaf color chart (LCC) or a soil plant analysis development (SPAD) chlorophyll meter, have shown promising results (Ladha et al., 2005); however, N management based on leaf color alone, without any consideration of crop biomass or photosynthetic rates, is considered to be the chief limitation of these tools. Fertilizer recommendation through these tools does not take into account the target or expected yield. In this context, the use of the optical sensor in agriculture for real-time N management, in which the total biomass or photosynthetic rates are considered, appears to be a promising option (Ma et al., 2001; Raun et al., 2001). Spectral vegetation indexes, such as the normalized difference vegetation index (NDVI), could successfully estimate the photosynthetic efficiency of the crop (Raun et al., 2001) and it is sensitive to the leaf area index and green biomass (Ma et al., 2001). By estimating the mid-season N requirement, the NDVI sensor could act as a framework for rational N management in cereals (Singh et al., 2011). A robust relationship between NDVI and grain yield showed higher NUEs than the conventional approach of N recommendation (Ali et al., 2015; Ali et al., 2020; Singha and Mitra, 2020). Considering the inadequacy of the general recommendation, this experiment has been planned to assess the impact on wheat of using NDVI sensor-based N management to ensure high yields and NUEs with minimum loss of N from the soil. We expect that N scheduling in wheat based on the NDVI sensor could improve yield performance and NUE through the improved synchronization of N application with proper growth stages, through which the sustainability of the rice–wheat system could be maintained.

## 2 Materials and methods

### 2.1 Experimental site

The study was conducted in the research field of Uttar Banga Krishi Viswavidyalaya (UBKV), Coochbehar, West Bengal, India (26°24′02.2″N, 89°23′21.7″E, 43 m above mean sea level) over two consecutive wheat seasons during 2017–18 and 2018–19. The layout was kept undisturbed for the entire experimental period (including the growing of rice crops between wheat seasons under uniform fertility levels). The pH of the experimental site was 5.78 with an organic carbon concentration of 0.83% and 188.16, 27.05, and 141.90 kg ha^–1^ of mineralizable nitrogen, available phosphorus, and available potassium, respectively.

### 2.2 Meteorological parameters during the crop growing period

**Figure 1:**
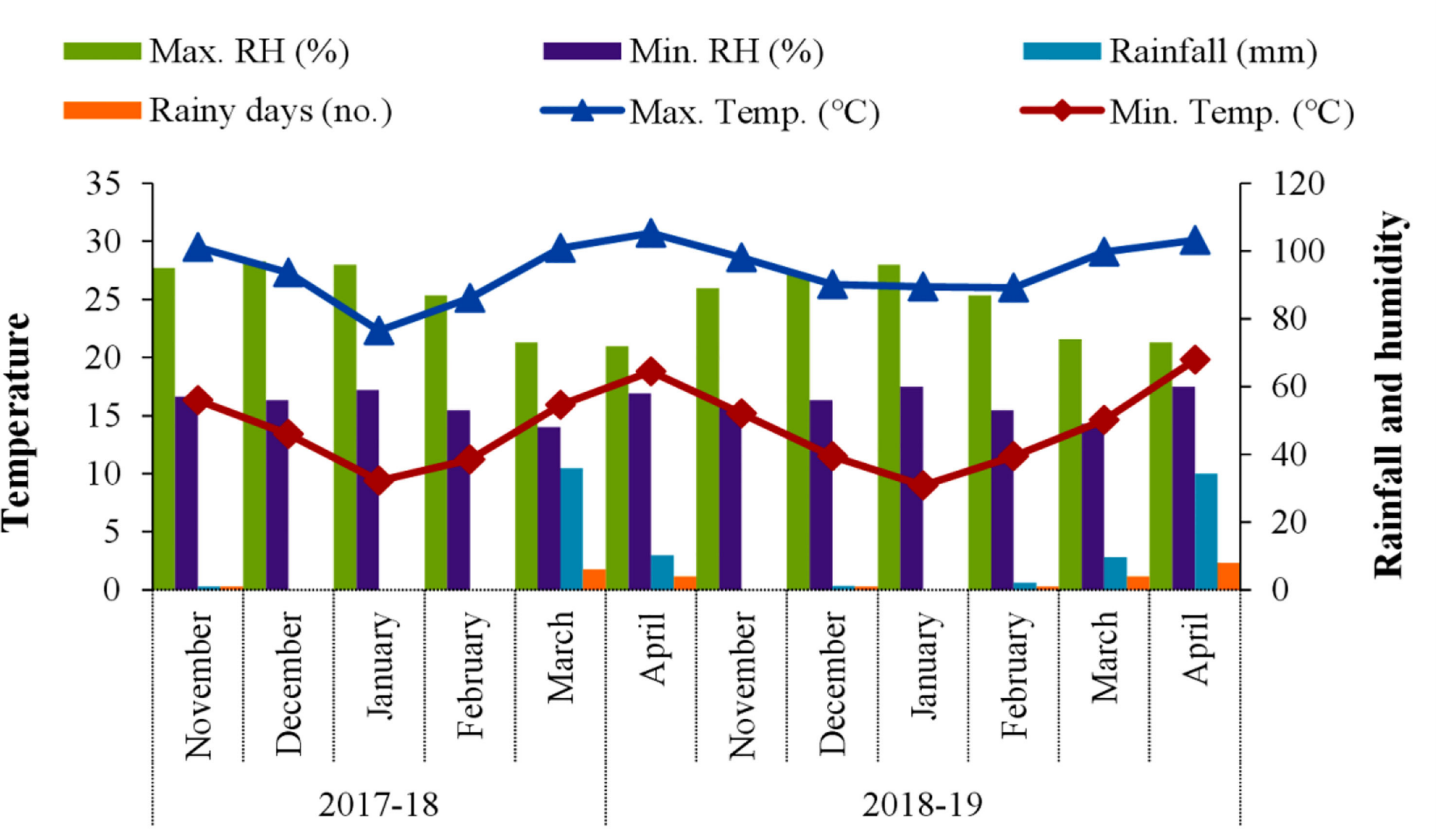
Prevailing meteorological data during the experiment.

The annual precipitation of this area varied between 2800 and 3000 mm with relatively dry winter months. Being a per-humid climate, relative humidity remained very high even during the winter months (above 90% from November to January and around 86%–87% during February). During crop season, there was little variation in the temperature during both years, and, overall, there was a favorable temperature regime for the crop. In general, the temperature showed a rising trend from mid-February and reached its peak during May. A notable amount of rainfall (i.e., 36 mm) was received in March 2018, whereas there was less rain in March 2019 (i.e., 9.62 mm). The prevailing meteorological parameters during the period of experimentation reflected a favorable year for wheat (**Figure 1** and **Table S1**).

### 2.3 Treatment details

The treatments and their combinations (**Table 1**) were arranged in a randomized complete block design and repeated three times. The size of each experimental plot was 8 m × 5.4 m. The detailed doses and splitting of N under various treatments, particularly the NDVI sensor-guided treatments, are presented in **Table 2**.

**Table 1:** Details of treatment.

Treatment	Dose (kg N ha^–1^)	Details
T1	No application	Absolute control (No N, P, or K)
T2	150	50% as basal + 25% CRI + 25% AT
T3	120	50% as basal + 25% CRI + 25% AT
T4	150	50% as basal + 50% CRI
T5	120	50% as basal + 50% CRI
T6	60 + NDVI	30 kg N each as basal and at CRI + NDVI* sensor
T7	90 + NDVI	30 and 60 kg N as basal and at CRI + NDVI sensor
T8	120 + NDVI	60 kg N each as basal and at CRI + NDVI sensor
T9	90 + NDVI	60 and 30 kg N as basal and at CRI + NDVI sensor
T10	225	50% basal + 50% at CRI

NDVI readings were taken at 45 and 65 days after seeding.

CRI, crown root initiation; AT, active tillering.

**Table 2:** Nitrogen application at various growth stages under different treatments.

Treatment	N application (kg ha^–1^)									
	2017–18					2018–19				
	Basal	21 DAS	45 DAS	65 DAS	Total	Basal	21 DAS	45 DAS	65 DAS	Total
T1	0	0	0	0	0	0	0	0	0	0
T2	75	37.5	37.5	0	150	75	37.5	37.5	0	150
T3	60	30	30	0	120	60	30	30	0	120
T4	75	75	0	0	150	75	75	0	0	150
T5	40	40	40	0	120	40	40	40	0	120
T6	30	30	44	15	119	30	30	42	16	118
T7	30	60	40	10	140	30	60	35	7	132
T8	60	60	35	0	155	60	60	32	0	152
T9	60	30	42	10	142	60	30	35	5	130
T10	112.5	112.5	0	0	225	112.5	112.5	0	0	225

Treatments detailed in **Table 1**.

DAS, days after seeding.

### 2.4 Crop management practices

The land preparation started with ploughing the site twice using a tractor-drawn cultivator. The soil was exposed would be more appropriate open for the next 10 days to reduce the soil moisture. Afterwards, a rotavator was used in a crisscross pattern to create a desirable tilth. In the second year, the land was prepared with the help of a power tiller within the plot area only without disturbing the previous layout. The crop received nitrogen as per the allocated treatment with 60 kg of P_2_O_5_ ha^–1^ and 40 kg of K_2_O ha^–1^ (P and K levels were uniform for all the treatments), as recommended by the All India Coordinated Research Project on Wheat for this zone. The entire quantity of P and K fertilizers was applied during the final land preparation, while the N fertilizers were applied according to the treatment schedule. Being pH of the soil indicates slight acidity, lime was applied once in 3 years as per the requirement and it was applied during land preparation for the rice crop (i.e., the preceding crop in the rotation). Wheat variety HD 2967, this variety is meant for timely sowing condition under irrigated ecosysytem with a potential yield of 6.5 t ha^–1^, was used in this experiment. This variety was developed by the Indian Agricultural Research Institute, New Delhi, India, and it has the pedigree ALONDRA/CUCKOO/URES-81/HD-2160-M/HD-2278[4251][4282]; HD-2733/K-9423/K-9351[4281]. After treating the seeds with carbendazim at 2.5 g kg^–1^, the seeds were line sown, maintaining 20-cm spacing between the lines, with a seed rate of 100 kg ha^–1^. After seeding, pre-emergence pendimethalin at 30% Emulsifiable Concentrate (EC) was applied at 1 kg ai ha^–1^ to keep the plots free from grassy weeds infestation; thereafter, carfentrazone-ethyl at 40% Dry Flowable (DF) was applied post emergence at 4 weeks after seeding at 20 g ai ha–1 to kill the broad-leaved weeds. There were two applications of boron, once at 35 days and, again, at 55 days after seeding, in the form of Solubor™ (B 20%) at 0.2%. Zn-EDTA 12% at 0.10% was also sprayed at 55 days after seeding. Four irrigations were applied during the crown root initiation (CRI), active tillering, jointing, and milking stages during both years. The check basin method of irrigation was followed, keeping the depth of irrigation at 5 cm. After threshing and drying, grain yield was recorded at 13% moisture.

### 2.5 Soil and plant analysis

The pH of the experimental soil was determined with a Sorensen’s pH meter (1909) using a soil–water suspension (1:2.5) by the potentiometric method. Mineralizable N of the soil was measured by the alkaline potassium permanganate method (Subbiah and Asija, 1956). Available phosphorus in the soil was measured by Bray’s method (Bray and Kurtz, 1945) with the help of a spectrophotometer at a wavelength of 660 nm. The available potassium in the soil was measured by the ammonium acetate extraction method, which involves extracting the soil with neutral 1(N) ammonium acetate (adjusted to pH 7.0), and the available potassium content was evaluated with the help of a flame photometer (Jackson, 1973). The available organic carbon was measured by the wet digestion method (Walkley and Black, 1934).

For plant analysis, grain and straw portions were crushed and kept separately with suitable descriptions and identification marks. The total N content in plants was determined by the modified micro-Kjeldahl method (Jackson, 1973). The P content of the plant materials was determined by triacid digestion through a vanadomolybdate–orthophosphate complex of yellow color in HNO_3_ medium with the help of a spectrophotometer at 420 nm wavelength and by using a standard curve (Jackson, 1973). The potassium content of the plant materials was measured by the triacid digestion method with the help of a flame photometer and by using a standard curve as described by Muhr et al. (1965). The total uptake of N by the wheat at harvest was determined on a dry weight basis by multiplying the total dry matter of the crop with its corresponding content of N. It is expressed in kg N ha^–1^.

### 2.6 Nitrogen use efficiency indices and partial nitrogen balance

The following NUE indices were calculated, as per the following formulae:

Partial factor productivity (kg grain kg^–1^ nitrogen applied) = yield under treatment (kg ha^–1^) amount of nitrogen added (kg ha^–1^). (1)
Agronomic N use efficiency (kg grain kg^–1^ nutrient applied over control) = [yield under test treatment (kg ha^–1^) – yield under control (kg ha^–1^)][unit of nitrogen applied in the treatment (kg ha^–1^)]. (2)
Agrophysiological efficiency (kg grain kg^–1^ nutrient uptake) = [(grain yield under fertilizer treatment (kg ha^–1^) – grain yield under control (kg ha^–1^)]/[(N uptake of nutrient in test treatment (kg ha^–1^) – N uptake of nutrient in control (kg ha^–1^)]. (3)
Apparent N recovery (%) = [(amount of nutrient taken from test treatment plot (kg ha^–1^) – the amount of nutrient taken from the control plot (kg ha^–1^)]/amount of nutrient added (kg ha^–1^). (4)
Economic N use efficiency (kg grain ₹^–1^ of investment in N) = yield under treatment (kg ha^–1^)/amount invested in nitrogen (₹ ha^–1^). (5)

The partial N-balanced approach provides a quantitative framework of N inputs and outputs. Nitrogen addition through both fertilizers and inherent soil N were taken as input. Similarly, N removal from grain and straw was considered as output. The expected N balance was worked out by subtracting the total removal (results) from total inputs (soil plus fertilizer N), and this predicted balance was further compared with the actual soil N status after harvest of the crop to express gain or loss of N (Rana et al., 2017).

### 2.7 Statistical analysis

Analysis of variance (ANOVA) was used for the randomized complete block design. The significance of various nitrogen scheduling treatments was tested by mean squared error as proposed by the Fisher–Snedecor *F*-test at a 5% probability level (Cochran and Cox, 1950; Panse and Sukhatme, 1967). A Fisher and Yates’s table was consulted for the computation of critical differences and comparisons. Finally, the mean values of each treatment were evaluated by Duncan’s multiple range test (DMRT) using IBM SPSS Statistics version 20.0.3 (IBM Corporation, Armonk, NY, USA). In addition, the relationships between the various NUE indices were assessed using bivariate correlation analysis (Pearson’s correlation coefficients and a two-tailed significance test).

## 3 Results

### 3.1 Changes in nitrogen scheduling influence the grain and straw yields as well as harvest index

Varying N scheduling had a significant effect on wheat grain yields in both years of the experiment (**Table 3**). The crops receiving 60 kg N ha^–1^ at baseline and 60 kg N ha^–1^ at CRI stages followed by NDVI-based N application (T8) achieved the maximum grain yield, followed by the crops receiving 75 kg N ha^–1^ at baseline along with 37.5 kg ha^–1^ each at the CRI and tillering stages. These two stages (i.e., CRI and active tillering) coincided with the timings of the second and third irrigation events.

**Table 3:** Grain yield, straw yield, and harvest index of wheat as influenced by varying nitrogen scheduling.

Treatment	Grain yield (t ha^–1^)		Straw yield (t ha^–1^)		Harvest index	
	2017–18	2018–19	2017–18	2018–19	2017–18	2018–19
T1	1.757a	1.353a	3.162a	2.382a	0.36b	0.36a
T2	4.725d	4.720d	6.850bc	6.603bc	0.41d	0.42b
T3	4.296bc	4.098b	6.951bc	6.265bc	0.38c	0.40b
T4	4.527cd	4.700d	6.690bc	6.910	0.40d	0.40b
T5	4.140b	4.125b	6.290b	6.395bc	0.40d	0.39b
T6	4.148b	4.030b	6.347b	5.924b	0.40d	0.40b
T7	4.523cd	4.355bc	6.921bc	6.740cd	0.40d	0.39b
T8	4.957d	5.068e	7.192d	7.084d	0.41d	0.42b
T9	4.450bc	4.385c	6.770bc	6.675bc	0.40d	0.40b
T10	4.513cd	4.637d	8.588e	8.763e	0.34a	0.35a
LSD (0.05)	**	**	**	**	**	**

Numbers followed by various lowercase letters within a column are significantly different from each other at a *p*-value ≤ 0.05 and are otherwise statistically on par; **significant at a 5% level of significance (*p* ≤ 0.05). LSD, least significant difference.

In the treatments in which Less nitrogen compared to 150 kg/ha (the optimum dose) was applied (i.e., T2, T5, and T6), the grain yield was recorded as lower than in treatments with higher N application doses. It was noted that higher N application during CRI stages followed by further application based on the NDVI sensor recorded better yields than lower doses of N application at CRI. The N-rich plot did not result in higher yields owing to the lower number of filled grains per spike (data not presented) despite having higher spikes per m^2^. However, the maximum straw yield was achieved in the N-rich plots in both years of the experiment. This was followed by treatment T8, in which 60 kg N ha^–1^ at baseline and 60 kg N ha^–1^ at CRI stages was applied, followed by the NDVI-based application of N. The harvest index varied significantly among the various N schedules, although there was little variation in the treatments receiving fixed or variable doses of N through the NDVI sensor with the exception of the absolute control (T1) and N-rich (T10) treatments, in which the harvest index value was much lower in both the years.

### 3.2 Nitrogen uptake by wheat grain and straw is influenced by changes in nitrogen scheduling

**Figure 2:**
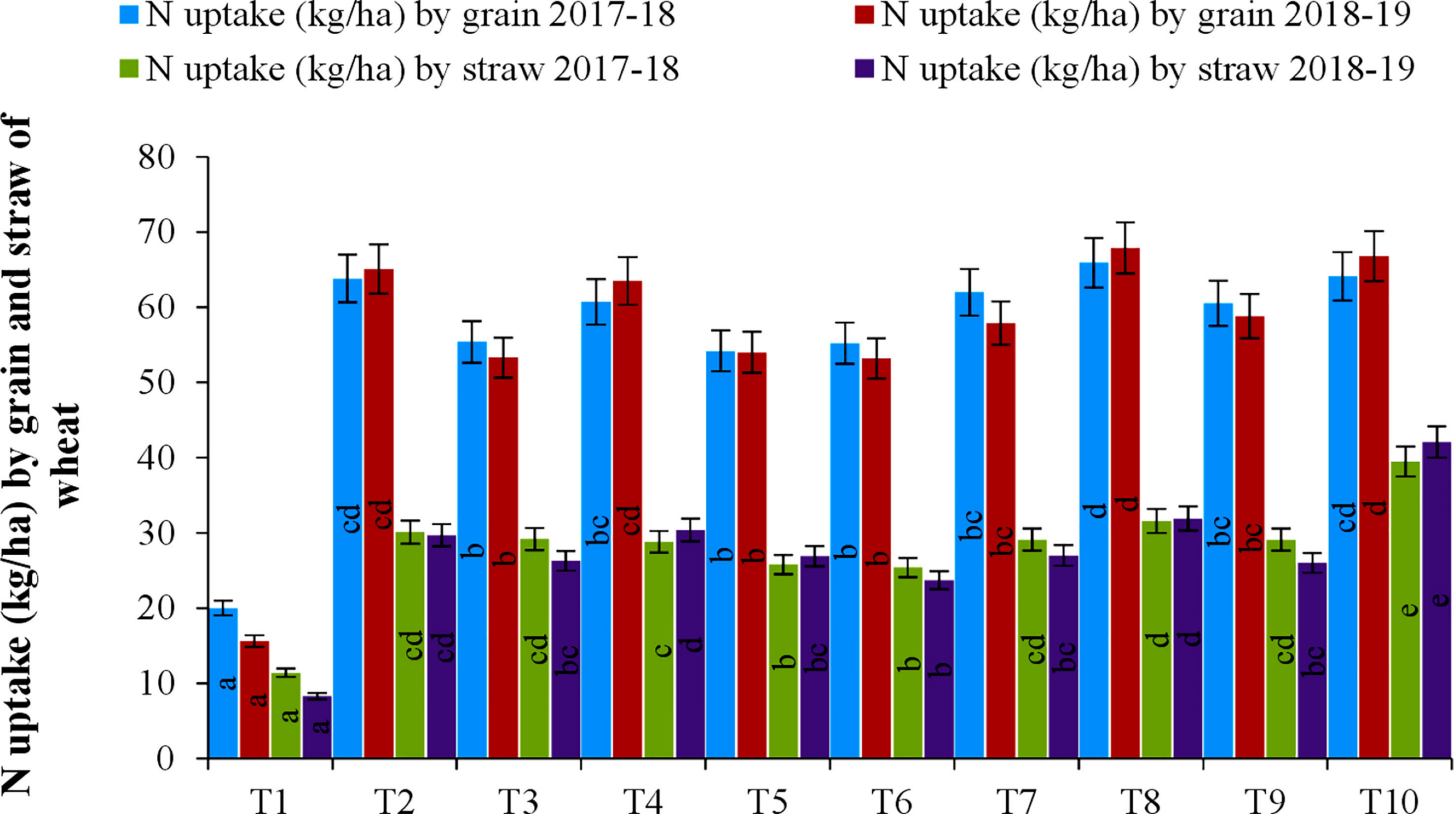
Nitrogen uptake by grain and straw of wheat, as influenced by various nitrogen schedules. Lowercase letters within a column are significantly different from each other at a *p*-value ≤ 0.05 and are otherwise statistically similar; **significant at a 5% level of significance (*p* ≤ 0.05).

The crops receiving 60 kg N ha^–1^ at baseline along with 60 kg N ha^–1^ at CRI stages followed by NDVI-based N application (T8) also achieved the highest N uptake in its grain (65.9 and 67.9 kg N ha^–1^ during the first and second year of the experiment, respectively). It was closely followed by the N-rich treatment (64.1 and 66.8 kg N ha^–1^ during 2017–18 and 2018–19, respectively). Despite a higher proportion of N in grain and straw in the N-rich treatment, the uptake was somewhat lower in T10 owing to its lower grain yield than T8 (**Figure 2** and **Table S2**). It was evident that there was little variation in grain N uptake between treatments T2, T4, T7, T8, T9, and T10. In contrast to grain N uptake, the straw N uptake was highest (39.5 and 42.1 N kg ha^–1^ during 2017–18 and 2018–19, respectively) in N-rich plots where 225 kg N ha^–1^ was applied in two equal doses, and was significantly higher than in any other treatment in the experiment (**Figure 2** and **Table S2**). The lowest grain and straw N uptake were seen the absolute control treatment (T1).

### 3.3 Nitrogen use efficiency indices are influenced by changes in nitrogen scheduling

Partial factor productivity of nitrogen (PFP_N_) and agronomic NUE showed a decreasing trend as the rate of N application increased. We observed a lower PFP_N_ and agronomic NUE as the dose of N increased from 120 to 150 kg N ha^–1^. The further increase in N rates from 150 to 225 kg N ha^–1^ in N-rich plots reflected the lowest N response regarding PFP_N_ and agronomic NUE. It was revealed that the lower rates of N, whether applied through fixed rates or at variable rates using NDVI, resulted in similar PFP_N_ and agronomic NUE values (**Table 4**). Irrespective of the number of splits, a dose of 120 kg N ha^–1^ showed the highest PFP_N_ and agronomic NUE values. As the expenditure towards N fertilizers was lower in treatments with lower rates of application, the economic N use efficiency (ENUE) value was also lower at 120 kg N ha^–1^ than other higher rates of N application. Invariably, the N-rich treatment (T10) showed the lowest ENUE value. It was evident that, except for in the N-rich plots where 225 kg ha^–1^ of N was applied, there was little variation in agrophysiological efficiency (APE) values among the treatments during both years of the experiment. The highest APE value was achieved with the treatment receiving 30 kg N ha^–1^ at baseline along with 30 kg N ha^–1^ at CRI stages, followed by NDVI-based N application (T6). Apparent N recovery (ANR) depends on the congruence between plant nitrogen demand and the quantity of nitrogen released from applied N. In general, ANR values were higher in treatments with lower N rates. It was interesting to note that T8 reflected the highest ANR value during the second year of study, indicating the feasibility of NDVI sensor-based N application.

**Table 4:** Nitrogen use efficiency indices in wheat as influenced by changes in nitrogen scheduling.

Treatment	Partial factor productivity of N (kg grain kg^–1^ of N)		Agronomic NUE (kg grain kg^–1^ of N)		Economic efficiency (kg grain ₹^–1^ of investment in N)		Agrophysiological efficiency (kg grain kg^–1^ of N uptake)		Apparent N recovery (%)	
	2017–18	2018–19	2017–18	2018–19	2017–18	2018–19	2017–18	2018–19	2017–18	2018–19
T1	**–**	**–**	**–**	**–**	–	–	–	–	–	–
T2	31.50bc	31.47b	19.79bc	22.45b	1.91b	1.91b	47.5bc	47.5b	41.7bc	47.3c
T3	35.80d	34.15d	21.16d	22.88b	2.17c	2.07c	47.7bc	49.3bc	44.3c	46.4bc
T4	30.18b	31.33b	18.47b	22.31b	1.83b	1.90b	47.7bc	47.8b	38.7b	46.6bc
T5	34.50cd	34.38d	19.86bc	23.10bc	2.09c	2.08c	49.0c	48.6bc	40.5bc	47.5c
T6	34.86cd	34.15d	20.09cd	22.69b	2.11c	2.07c	48.6c	50.5c	41.3bc	44.9b
T7	32.31bc	32.99bc	19.76bc	22.74b	1.96b	2.00bc	46.4b	49.2bc	42.6c	46.2bc
T8	31.98bc	33.34bc	20.65cd	24.44c	1.94b	2.02bc	48.4bc	49.0bc	42.7c	49.9c
T9	31.34b	33.73bc	18.96b	23.32bc	1.90b	2.04bc	46.2b	49.8bc	41.0bc	46.8bc
T10	20.06a	20.61a	12.25a	14.60a	1.22a	1.25a	38.2a	38.7a	32.1a	37.7a
LSD (0.05)	**	**	**	**	**	**	**	**	**	**

Numbers followed by various lowercase letters within a column are significantly different from each other at a *p*-value ≤ 0.05 and are otherwise statistically on par; **significant at a 5% level of significance (*p* ≤ 0.05). LSD, least significant difference.

### 3.4 Soil nitrogen status after the harvest of each crop is influenced by changes in nitrogen scheduling

Soil N status was estimated twice in each year, i.e., at the end of the wheat crop harvest and the end of the rice crop harvest (taken in the rotation after the wheat), and the changes in soil N status after each crop in the rotation are presented in **Table 5**.

**Table 5:** Soil nitrogen status after each crop in the rotation was influenced by various N schedules.

Treatment	Mineralizable N in soil (kg N ha^–1^)			
	2017–18		2018–19	
	End of wheat crop	End of rice crop	End of wheat crop	End of rice crop
T1	170.53	160.42	154.95	143.53
T2	185.38	172.31	181.73	170.25
T3	177.53	168.24	162.51	150.37
T4	182.28	170.25	178.38	166.23
T5	172.94	161.00	165.48	148.73
T6	175.32	160.81	157.31	145.32
T7	178.90	162.35	162.48	154.31
T8	184.00	170.52	172.30	160.38
T9	175.63	158.38	160.48	145.20
T10	192.72	187.34	196.29	188.75
Initial	188.16		171.23	

The initial N status (188.16 kg N ha^–1^) decreased in all treatments except in the N-rich strip, where there was a marginal increase in soil N (192.72 kg ha^–1^) after the wheat crop harvest in the rotation. Owing to the changes in N scheduling in wheat, it was seen that even with recommended doses of N application (150 kg N ha^–1^), the N status of the experimental soil decreased slightly. Even in T8, where 152–155 kg N ha^–1^ was applied based on the NDVI sensor, the soil N concentration decreased. In the treatment where no N was used, the decrease in soil N was the greatest (170.53 kg N ha^–1^). After the rice harvest (taken after wheat in the rotation), the N status in all treatments (including the control and N-rich treatments) further decreased by 10–17 kg N ha^–1^. After the second-year wheat crop harvest, the soil N increased slightly (by 2–9 kg N ha^–1^) in treatments where 150 kg N ha^–1^ or more was applied. For the remaining treatments, the soil N status decreased further, following the trend of the first year.

### 3.5 Partial nitrogen balance is influenced by changes in nitrogen scheduling

A statement of nitrogen inputs and outputs was expressed through the partial N-balanced approach. The sustainability of a system is examined through the retention of N in the soil. Although N inputs include direct addition through manures and fertilizers, atmospheric deposition, irrigation water, biological N fixation, etc., we have used only N addition through fertilizers and soil-inherent N as inputs. Similarly, we have concentrated on N removal through crop uptake only, although N is also removed through leaching, denitrification, volatilization, etc., which is difficult to measure. The N balance sheet was calculated after the wheat crop harvest in both years. During 2017–18, N balance was recorded as negative for all treatments except the control (which showed a positive balance), which reflected an overall loss of soil N under the various treatments, excluding the control. However, there was variation in the extent of N loss: the smallest losses were achieved when lower doses of N (118–120 kg N ha^–1^) were used. The greatest losses (–116.84 kg ha^–1^) were recorded for T10, the N-rich treatment (**Table 6**). The treatment in which no N was added recorded a positive balance (+13.77 kg N ha^–1^) after the first-year harvest of the wheat crop. During 2018–19, the N balance sheet showed a similar trend to the first year, with a negative balance for all treatments except the control.

**Table 6:** Nitrogen balance sheet in wheat as influenced by changes in N scheduling in 2017–18 and 2018–19.

Treatment	Initial soil N (kg N ha^–1^) (a)	Fertilizer N (kg N ha^–1^) (b)	Total N (kg N ha^–1^) (c = a + b)	Total grain + straw N uptake (kg N ha^–1^) (d)	Expected balance (kg N ha^–1^) (e = c – d)	Final balance (kg N ha^–1^) (f)	Net gain or loss (kg N ha^–1^) (g = f – e)
2017–18							
T1	188.16	0	188.16	31.4	156.76	170.53	+13.27
T2	188.16	150	338.16	93.9	244.26	185.38	–58.88
T3	188.16	120	308.16	84.6	223.56	177.53	–46.03
T4	188.16	150	338.16	89.4	248.76	182.28	–66.48
T5	188.16	120	308.16	80.0	228.16	172.94	–55.22
T6	188.16	119	307.16	80.6	226.56	175.32	–51.24
T7	188.16	140	328.16	91.0	237.16	178.90	–58.26
T8	188.16	155	343.16	97.6	245.56	184.00	–61.56
T9	188.16	142	330.16	89.6	240.56	175.63	–64.93
T10	188.16	225	413.16	103.6	309.56	192.72	–116.84
Treatments	2018–19						
T1	171.23	0	171.23	23.9	147.33	154.95	+17.62
T2	171.23	150	321.23	94.8	226.43	181.73	–44.70
T3	171.23	120	291.23	79.6	211.63	162.51	–49.12
T4	171.23	150	321.23	93.6	227.23	178.38	–48.95
T5	171.23	120	299.23	80.9	210.33	165.48	–44.85
T6	171.23	119	289.23	76.9	212.33	157.31	–55.02
T7	171.23	140	303.23	84.9	218.33	162.48	–55.85
T8	171.23	155	328.23	99.9	223.33	172.30	–51.03
T9	171.23	142	301.23	84.8	2.6.43	160.48	–55.95
T10	171.23	225	396.23	108.8	287.43	196.29	–91.14

### 3.6 The correlation matrix

Correlation studies were performed with several NUE indices, viz., PFP_N_, Agronomic Nitrogen Use Efficiency (ANUE), APE, ANR, and ENUE for both years (**Table 7**). The correlation matrix for 2017–18 and 2018–19 suggests a robust positive correlation among the various NUE indices (**Table 7**). It was observed that an absolute positive correlation existed between PFP_N_ and ENUE (*r* = 1) during both years of investigation. Apart from these two, the *r*-values for the other NUE indices ranged from 0.845 to 0.964 and from 0.845 to 0.985 in 2017–18 and 2018–19, respectively, indicating that the indices were highly correlated. As all these indices were calculated with respect to either grain yield, total biomass production, or total N intake and N rate, any change in N application rate primarily affected all these indices, for which the values showed a high positive correlation.

**Table 7:** Correlation matrix of various nitrogen use efficiency (NUE) indices during 2017–18 and 2018–19.

Index	2017–18				
	PFP_N_	ANUE	APE	ANR	ENUE
PFP_N_	1.00				
ANUE	0.963^**^	1.00			
APE	0.932^**^	0.947^**^	1.00		
ANR	0.917^**^	0.972^**^	0.845^**^	1.00	
ENUE	1.000^**^	0.964^**^	0.932^**^	0.918^**^	1.00
Characters	2018–19				
PFP_N_	1.00				
ANUE	0.967^**^	1.00			
APE	0.985^**^	0.958^**^	1.00		
ANR	0.875^**^	0.963^**^	0.845^**^	1.00	
ENUE	1.000^**^	0.967^**^	0.985^**^	0.876^**^	1.00

** Correlation is significant at the 0.01 level (two-tailed).

## 4 Discussion

### 4.1 Changes in nitrogen scheduling influence the grain and straw yields as well as harvest index

This study demonstrated that the crops receiving 60 kg N ha^–1^ at baseline along with 60 kg N ha^–1^ at CRI stages followed by NDVI sensor-based N application (T8) achieved the highest grain yield, although the highest straw yield was obtained in N-rich plots where 225 kg N ha^–1^ was applied. Excessive nitrogen application in the N-rich treatment triggered vegetative growth, and very often depressed the activity of superoxide dismutase and peroxidase and increased the accumulation of reactive oxygen species (ROS) and malondialdehyde. Higher NDVI values at anthesis interfered with N and lipid metabolism through increased lipid peroxidation, thus leading to poor grain filling in wheat (Kong et al., 2017). The activity of certain flag leaf enzymes, which play a crucial role in grain filling, is also disrupted under excess nitrogen application, reducing the number of filled grains (Kong et al., 2017). Singh et al. (2011) observed robust relationships between N application at Feekes’s growth stages 5 and 6 and 7 and 8 and actual wheat yields.

In our experiment, the NDVI-based N application coincided with Feekes’s growth stages 5 and 6 [45 days after seeding (DAS)] and 7 and 8 (65 DAS). The yield was enhanced, compared with a fixed nitrogen application dose, when N was applied based on the NDVI sensor at 45 and 65 DAS. Our study showed that the crop did not require any application of N at 65 DAS if 60 kg ha^–1^ of N was applied at baseline during the final land preparation stage and again at the CRI stage. Our results indicated that the timing of the nitrogen application and the quantity of nitrogen involved in each split determined the yield. A higher number of divisions may not always result in higher yields if the amount is insufficient during these crucial growth stages. Sobh et al. (2000) and Melaj et al. (2003) also demonstrated an increase in yield with the synchronization of nitrogen application at essential growth stages. Mitra et al. (2014) reported that grain yield was significantly affected by different N rates owing to the effect on spikelets per spike, grains per spike, and 1,000-grain weight.

### 4.2 Nitrogen uptake by grain and straw of wheat is influenced by changes in nitrogen scheduling

The higher N concentrations in the grain from plots with more applied N may be attributed to a better response to applied N through the translocation of absorbed N to grain. N supply in the soil may also affect the grain N concentration in combination with genetic variability (Lopez-Bellido et al., 2004). Achieving good yields with higher N concentrations in grain resulted in higher grain N uptake in treatment T8. Sinebo et al. (2004) also reported higher N concentrations in wheat grains with increasing N supply. However, the straw yield in the N-rich treatment was far higher than in any other treatments in the experiment. The straw N concentration was also higher in the N-rich treatment, as was the total straw N uptake. Nitrogen uptake depends on the timing of its application (Kumar and Yadav, 2005). Split application of N after the first irrigation significantly increased the N uptake, and N application at critical stages of crop growth resulted in higher yields and, therefore, higher N uptake. Mattas et al. (2011) observed a significant increase in N uptake in three-split applications over two-split applications in Ludhiana, India. Through leaf measurements using an NDVI sensor, N uptake as well as grain yields could be improved by a considerable amount (Ali et al., 2015). Thus, the NDVI sensor could act as a framework for refining nitrogen management in wheat.

### 4.3 Nitrogen use efficiency indices are influenced by changes in nitrogen scheduling

Most NUE indices showed higher values with lower rates of N application. Increasing yields in wheat with lower PFP_N_ was previously reported by Haile et al. (2012) and Mondal et al. (2018). These values indicate a higher proportion of yield attainment per unit of N application using lower rates of N application. Our study suggests that the application of N based on an NDVI sensor could be more efficient than a fixed rate of N application applied with two or three splits. Better synchronization, with the timely application of N at the proper doses, may be the key to NDVI-based N applications. Raun et al. (2002) showed that NUEs in wheat were improved by more than 15% when N fertilization was based on an in-season estimate of yield (INSEY) calculated from an optically measured NDVI. PFP_N_ and agronomic NUE are essential tools for judging N management’s nutrient use efficiency and developing environmentally sound nutrient management strategies. Partial factor productivity increases with precise fertilizer application, better crop management practices, and an increased nutrient conversion ratio in plant systems. In the treatments in which N was applied based on the NDVI sensor (T6–T9), there was a slight variation in ENUE. More importantly, these values were statistically on par with the highest values recorded during 2018–19. Regarding economic N use efficiency (ENUE), higher values were recorded in treatments with lower rates of N application than higher rates. This indicates that investment in N was lower in those treatments for which there was an increased ENUE value, owing to the lesser use of N. The yields obtained with higher N doses may not always be economically feasible. These indices suggest that the use of an NDVI sensor could have the potential to achieve both higher yields and increased ENUE values. Biermacher et al. (2006) showed significant nitrogen savings under variable-rate N applications. Higher ENUE values have been reported in wheat using site-specific nutrient management practices with precise N application in the sub-Himalayan plains of West Bengal, India (Mondal et al., 2018; Mitra et al., 2019).

As APE is related to grain yield only, it was a powerful tool in assessing the effect of N application on grain yields. Here also, the values were slightly higher in treatments with less applied N, although there was some slight variation. Achieving higher grain yields with lower rates of N application brought about an increase in APE values under both fixed and (using the NDVI sensor) variable rates of N application. There was no evidence that the APE values increased steadily with an increasing number of splits. Higher APE values in wheat with lower rates of N application have been previously reported (Gauer et al., 1992).

In general, the apparent nitrogen recovery (ANR) of crops was higher in treatments receiving less N. From this trend, it was evident that the N-rich plots recorded the lowest ANR values. It was further noted that the ANR values were comparable in NDVI-based N application treatments with those of lower-N treatments. It is interesting to note that T8 recorded the highest ANR value (49.9%) in 2018–19, reflecting more effective N splitting in terms of a higher N uptake value (comparable with N-rich plots). This suggests that site-specific adjustments to nutrient management guidelines could achieve higher yield performances and an increase in profit.

The correlation matrix in both seasons suggested a robust positive correlation among the various NUE indices (**Table 7**). An absolute positive correlation was observed between PFP_N_ and ENUE, (*r* = 1). Apart from these two, the *r*-values for the other NUE indices ranged from 0.845 to 0.964 and from 0.845 to 0.985 in 2017–18 and 2018–19, respectively, indicating that the indices were highly correlated. As all these indices were calculated with regard to grain yield, total biomass production, or total N intake and N rate, any change in N rate primarily affected all these indices, for which the values showed a high positive correlation. The findings of our current study in relation to NUE indices were also confirmed by several earlier studies (Fageria and Baligar, 2005; Chuan et al., 2016), which confirmed that the application of higher amounts of fertilizer N than the required quantities leads to high N losses and low NUEs. On the other hand, other findings stated that a site-specific nutrient management strategy based on synchronizing fertilizer N supply with the N demand of the crop and supply of N from soil sources could cause soil N to increase, as confirmed by Ali et al. (2015) and Ali et al. (2020), who also reported that the use of an NDVI sensor could optimize the N dose to improve NUEs with reduced environmental risks.

### 4.4 Soil nitrogen status after the harvest of each crop is influenced by changes in nitrogen scheduling

Soil N status decreased after the wheat harvest, even with the application of 150 kg N ha^–1^ to the crop. The N status of the experimental soil suggests that N scheduling in the wheat–rice rotation has to be restructured more precisely to maintain the overall N status of the soil and the sustainability of the system. In fact, N application of 150 kg N ha^–1^ or more in wheat did not decrease the overall N status of the experimental soil to a greater extent than lower-N applications, as reflected in the data after the wheat harvest. However, after the rice harvest, the decrease in soil N was very high. As there was no treatment difference in the rice crops concerning N nutrition, the extent of the decrease in soil N status was similar across all treatments. While studying the rice–wheat system in the Tailake region of China, Xue et al. (2014) noted that increased fertilizer N input was primarily responsible for the low NUE. The authors also found higher losses after the rice harvest, possibly owing to higher losses of N through leaching and runoff. N loss could be minimized with reduced chemical inputs combined with organic inputs. We feel that a more thorough understanding of N dynamics in this rotation is required to maintain the system’s sustainability.

### 4.5 Partial nitrogen balance is influenced by changes in nitrogen scheduling

The partial N balance showed that the extent of N losses was slightly smaller in the second year than in the first year. The greatest negative balance was recorded in the N-rich plot (–91.14 kg N ha^–1^). The control treatment recorded a positive balance (+7.62 kg N ha^–1^) after the harvest of the second-year wheat. The negative balance indicated that the soil itself is a source of (and not a drain on) N—a result of increased mineralization of crop residues and the conversion of organic N into inorganic N, which has taken up the plants. N balance studies are always beneficial for long-term experiments (Ross et al., 2008; Pieri et al., 2011). Long-term nutrient management significantly affected the N-uptake and NUE indices. The reduced N losses combined with balanced N management could improve productivity with a lesser degree of environmental pollution (Dhawan et al., 2021). Minimum loss of N was previously reported in some studies in which organic manures were used with NPK fertilizers (Dhawan et al., 2021; Fazal et al., 2022). There are complexities in measuring a range of inputs and outputs and, depending on the climate and soil features, environments can undergo long-term change in just a few years (Watson and Atkinson, 1999). Sainju (2017) advocated that balancing N over 5-year cycles is highly beneficial for managing N.

## 5 Conclusions

From this 2-year field trial, it can be concluded that applying the normalized difference vegetation index sensor could be an essential tool for the rational management of fertilizer nitrogen in wheat grown in eastern sub-Himalayan plains. A dose of 60 kg N ha^–1^ at baseline and 60 kg N ha^–1^ at crown root initiation followed by nitrogen application controlled by the sensor (32–35 kg N ha^–1^) resulted in a significant improvement in yield parameters and nutrient uptake, although lower nitrogen doses (118–120 kg ha^–1^) resulted in higher values of various NUE parameters. Apparent nitrogen recovery values were comparable between sensor-based nitrogen application treatments and treatments receiving lower N doses. Despite the application of 150–155 kg N ha^–1^ in wheat, a negative balance of soil nitrogen suggested the need for further precision in nitrogen scheduling to maintain the system’s sustainability for this region.

## Data availability statement

The raw data supporting the conclusions of this article will be made available by the authors, without undue reservation.

## Author contributions

Conceptualization: BM, PS, AR, and AS; methodology: BM, PS, AR, and AS; formal analysis: MS, MB, SA, and AH; data curation: MS, MB, SA, and AH; statistical expertise: MS, MB, SA, and AH; writing—original draft preparation: MS, MB, SA, and AH; writing—review and editing: SA and AH; visualization: BM, PS, AR, and AS; supervision: BM and AS; and funding acquisition: SA and AH. All authors contributed to the article and approved the submitted version.
