# Supplementary material for: Normalized difference vegetation index sensor-based nitrogen management in bread wheat (Triticum aestivum L.): Nutrient uptake, use efficiency, and partial nutrient balance
Source: Front Plant Sci. 2023 Apr 4;14:1153500. doi: 10.3389/fpls.2023.1153500 (PMC10111010; doi:10.3389/fpls.2023.1153500)
Supplement: Supplementary file 1 [file Table_1.docx]

**Table S1.** Meteorological data prevailed during the experimentation

| **Year** | **Months** | **Max. Temp. (℃)** | **Min. Temp. (℃)** | **Max. RH (%)** | **Min. RH (%)** | **Rainfall (mm)** | **Rainy days (no.)** |
| --- | --- | --- | --- | --- | --- | --- | --- |
| 2017-18 | November | 29.5 | 16.3 | 95 | 57 | 1 | 1 |
|  | December | 27.3 | 13.4 | 97 | 56 | 0 | 0 |
|  | January | 22.3 | 9.4 | 96 | 59 | 0 | 0 |
|  | February | 25.1 | 11.2 | 87 | 53 | 0 | 0 |
|  | March | 29.4 | 15.9 | 73 | 48 | 36 | 6 |
|  | April | 30.7 | 18.8 | 72 | 58 | 10.18 | 4 |
| 2018-19 | November | 28.6 | 15.2 | 89 | 54 | 0 | 0 |
|  | December | 26.3 | 11.5 | 93 | 56 | 1.16 | 1 |
|  | January | 26.1 | 9 | 96 | 60 | 0 | 0 |
|  | February | 26 | 11.5 | 87 | 53 | 2.23 | 1 |
|  | March | 29.1 | 14.6 | 74 | 49 | 9.62 | 4 |
|  | April | 30.1 | 19.8 | 73 | 60 | 34.34 | 8 |

**Table S2.** Nitrogen uptake by grain and straw of wheat as influenced by various nitrogen scheduling.

| **Treatments** | **Total grain N uptake (kg ha^-1^)** | | **Total straw N uptake (kg ha^-1^)** | |
| --- | --- | --- | --- | --- |
|  | **2017-18** | **2018-19** | **2017-18** | **2018-19** |
| T1 | 20.0a | 15.6a | 11.4a | 8.3a |
| T2 | 63.8cd | 65.1cd | 30.1cd | 29.7cd |
| T3 | 55.4b | 53.3b | 29.2cd | 26.3bc |
| T4 | 60.7bc | 63.5cd | 28.8c | 30.4d |
| T5 | 54.2b | 54.0b | 25.8b | 26.9bc |
| T6 | 55.2b | 53.2b | 25.4b | 23.7b |
| T7 | 62.0bc | 57.9bc | 29.1cd | 27.0bc |
| T8 | 65.9d | 67.9d | 31.6d | 31.9d |
| T9 | 60.5bc | 58.8bc | 29.1cd | 26.0bc |
| T10 | 64.1cd | 66.8d | 39.5e | 42.1e |
| LSD (0.05) | ** | ** | ** | ** |

Numbers followed by various lowercase letters within a column are significantly different from each other at p ≤ 0.05 and are otherwise statistically on par; **significant at a 5% level of significance (p ≤ 0.05)
